# Autologous blood patch intraparenchymal injection reduces the incidence of pneumothorax and the need for chest tube placement following CT-guided lung biopsy: a systematic review and meta-analysis

**DOI:** 10.1186/s40001-024-01707-9

**Published:** 2024-02-09

**Authors:** Teng Li, Qiang Zhang, Wenjun Li, Yun Liu

**Affiliations:** 1 Department of Interventional Radiology, The People’s Hospital of Weifang, 151 Guangwen Street, Weifang, 261041 Shandong China; 2 Department of Nuclear Medicine, The People’s Hospital of Weifang, 151 Guangwen Street, Weifang, 261041 Shandong China; 3 Department of Hematology, The People’s Hospital of Weifang, 151 Guangwen Street, Weifang, 261041 Shandong China

**Keywords:** Autologous blood patch, Lung biopsy, Pneumothorax, Chest tube

## Abstract

**Purpose:**

To assess the effectiveness of autologous blood patch intraparenchymal injection during CT-guided lung biopsies with a focus on the incidence of pneumothorax and the subsequent requirement for chest tube placement.

**Methods:**

A comprehensive search of major databases was conducted to identify studies that utilized autologous blood patches to mitigate the risk of pneumothorax following lung biopsies. Efficacy was next assessed through a meta-analysis using a random-effects model.

**Results:**

Of the 122 carefully analyzed studies, nine, representing a patient population of 4116, were incorporated into the final analysis. Conclusion deduced showed a noteworthy reduction in the overall incidence of pneumothorax (RR = 0.65; 95% CI 0.53–0.80; *P* = 0.00) and a significantly decline in the occasion for chest tube placement due to pneumothorax (RR = 0.45; 95% CI 0.32–0.64; *P* = 0.00).

**Conclusions:**

Utilizing autologous blood patch intraparenchymal injection during the coaxial needle retraction process post-lung biopsy is highly effective in diminishing both the incidence of pneumothorax and consequent chest tube placement requirement.

**Supplementary Information:**

The online version contains supplementary material available at 10.1186/s40001-024-01707-9.

## Introduction

Computed tomography (CT)-guided lung biopsy is a well-established and widely used technique essential for diagnosing lung lesions, with a diagnostic accuracy ranging from 88 to 97% [1]. The increasing prevalence of lung cancer, enhanced detection rates of asymptomatic lung nodules, and increased demand for tissue sampling for advanced molecular profiling and genomic analysis have contributed to the widespread use of this technique [2]. Although generally considered safe, it is an invasive procedure that carries certain risks. Pneumothorax, with an incidence of 10% to 45%, is the most commonly reported complication [3–5]. Patients with mild pneumothorax typically do not experience chest compression, and the pneumothorax is gradually absorbed, obviating the need for further treatment. However, in 1% to 15% of cases, severe pneumothorax necessitates chest tube placement, often leading to prolonged hospital stays and substantial economic consequences [6, 7].

Pneumothorax occurs when air escapes from the lung through the needle puncture site on the visceral pleura after withdrawing the coaxial needle [8]. To mitigate the risk of pneumothorax, several methods have been proposed to seal the biopsy tract upon needle withdrawal, using various materials. The autologous blood patch is particularly noteworthy, frequently mentioned due to its simplicity, cost-effectiveness, and lack of identified safety risks.

The current literature presents inconsistent findings regarding the efficacy of autologous blood patch injections. Initial studies reported no significant statistical differences in pneumothorax rates associated with autologous blood patches. However, these studies used a C-arm fluoroscopic control for monitoring the biopsy procedure, which has since been superseded by CT-guided techniques [9, 10]. A randomized-controlled trial (RCT) conducted by Lang et al. involving 100 participants demonstrated the method's effectiveness in reducing both pneumothorax incidence and the need for chest tube placement [11]. Conversely, Malone et al.’s separate RCT, which included 242 participants, observed a significant reduction in pneumothorax requiring chest tube placement, but the overall decrease in pneumothorax incidence was not statistically significant [12]. Several other cohort studies have reported similarly inconclusive results [13–19]. This variability in findings likely explains why the only official guideline dedicated to CT-guided lung biopsy did not endorse intraparenchymal autologous blood patch use and questioned its practicality [20].

Therefore, we conducted a systematic review and meta-analysis to synthesize the existing evidence and evaluate the efficacy of autologous blood patch intraparenchymal injections during CT-guided lung biopsies, specifically concerning the incidence of pneumothorax and the need for chest tube placement.

## Materials and methods

The present systematic review and meta-analysis followed the protocol set forth by all authors, and adhering to the 2020 statement of Preferred Reporting Items for Systematic Reviews and Meta-Analyses (PRISMA) [21]. Registration of the study protocol was promptly accomplished on PROSPERO (www.crd.york.ac.uk/prospero/), bearing the registration number CRD42023468343. Given that all analyses originate from previously published studies, the requisition for ethical approval and patient consent was deemed superfluous.

### Literature search and study selection

Two authors, Teng Li and Yun Liu, independently executed an exhaustive search across various databases include PubMed, EMbase, Cochrane Library, Web of Science, and ClinicalTrials.gov. This search included studies published until September 20, 2023. The primary keywords used in our search strategy encompassed "autologous blood", “blood patch”, “pneumothorax”, and “biopsy” in all conceivable combinations. Furthermore, we scrutinized the reference lists of the identified articles to uncover any additional eligible studies for inclusion in our analysis.

This meta-analysis included studies that met the following inclusion criteria: (1) the use of autologous blood patch to reduce the risk of pneumothorax after lung biopsies, and (2) at least one quantifiable outcome. Conversely, studies were excluded if they met any of the following criteria: (1) they were animal studies, (2) biopsies were not performed under CT guidance, or (3) maneuvers other than the autologous blood patch (e.g., biopsy-side-down positioning and rapid rollover) differed between the two groups.

Authors Qiang Zhang and Wenjun Li executed a detailed examination of titles and abstracts derived from database searches. When a title and abstract were deduced to align with the inclusion criteria by either author, they advanced to acquire the full-text version of the respective articles. The decision to include or exclude an article arose from a consensus between both authors, with disagreements resolved via discussion or, when necessary, consultation with a third author, Yun Liu.

### Data extraction

For data extraction, the authors jointly devised a data extraction sheet. To test its dependability, a preliminary trial was performed on two articles. Individual data extractions were carried out by Qiang Zhang and Wenjun Li, and were mutually reviewed for data accuracy. Disputes, if any, were resolved by engaging in discussions or by seeking input from a third author, Yun Liu. The extraction procedure resulted in a range of data elements, encapsulating the basic features of the included studies like authors, year of publication, location, and design of the study, in addition to patient-specific facets such as the diameters of guiding and biopsy needles used, guidance method, sample size, average patient age, location, and size of lesions. Data outcomes compiled encompassed pneumothorax episodes and instances requiring chest tube placements in both intervention and control groups. This meta-analysis utilized only reported data, and no assumptions were made for missing or unclear information.

### Quality assessment

The methodology quality of the integrated RCTs was assessed via the modified Jadad score, bifurcating them into low-quality (scores of 1–3) and high-quality (scores of 4–7) studies. Concurrently, cohort studies underwent scrutiny through the 9-star Newcastle–Ottawa Scale (NOS), rendering the quality classifications as high (scores of 7–9), medium (scores of 4–6), or low (scores below 4). Two independent reviewers, Teng Li and Yun Liu, investigated these studies, the final decision of which hinged on a consensus.

### Statistical analysis

The Risk Ratio (RR) for the incidence of pneumothorax and chest tube placement, inclusive of 95% confidence intervals (CI), was calculated utilizing a random-effects model. Statistical heterogeneity was measured using I^2^ statistic and I^2^ statistic greater than 50% indicates significant heterogeneity. The sources of heterogeneity were explored using the Galbraith plots. A sensitivity analysis, executed by sequentially excluding one study, determined stability. Subgroup analysis was conducted by the study design, guidance method, and blood status. The potential publication bias was assessed visually with a funnel plot and quantified with Egger’s test to ascertain funnel plot asymmetry, with a P value less than 0.05 signaling significant publication bias. Stata version 16 (Stata 16 software, Stata Corp, College Station, Texas, USA) was used for all data analyses.

## Results

### Literature search, study characteristics, and quality assessment

**Fig. 1:**
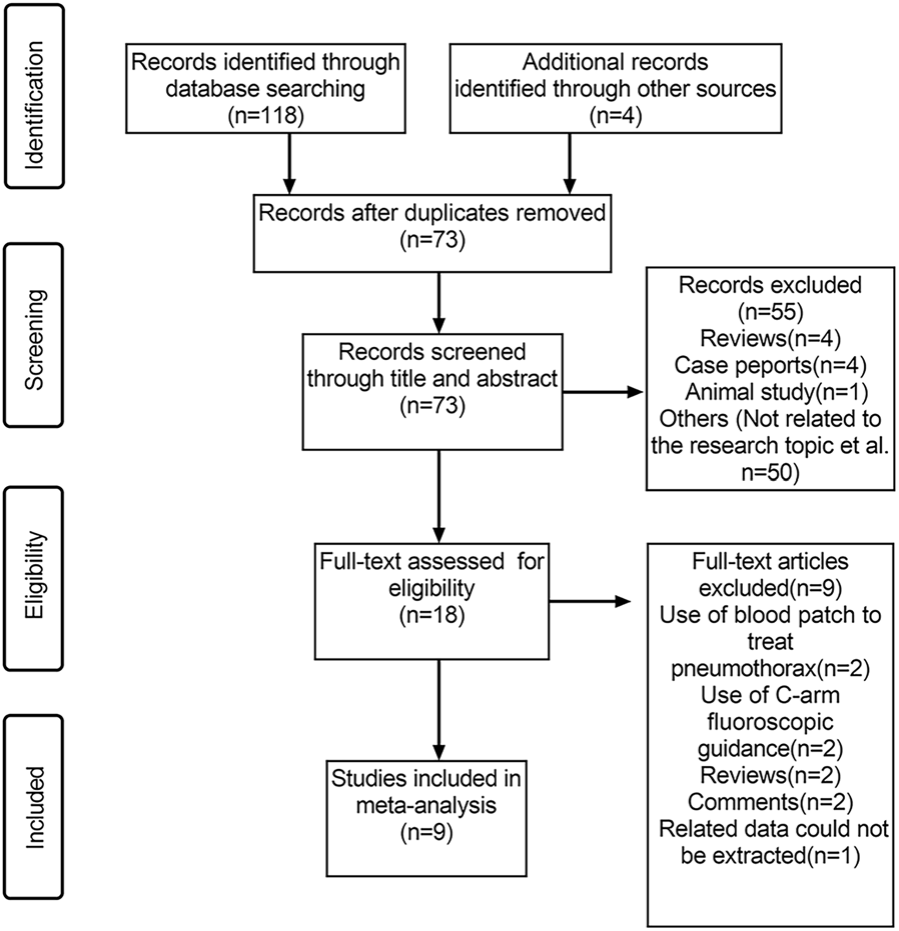
Flowchart of the study selection

A meticulous search across several databases and reference lists taken from identified articles, rendered an initial total of 122 articles. Upon duplicate removal, the total was winnowed down to 73 unique articles. A further cull ensued, taking into account reviews, case reports, animal studies, or content deemed irrelevant to this research, leading to the exclusion of 55 articles. In applying the pre-established eligibility criteria, nine additional full-text articles were disqualified, leaving us with a final total of nine articles that were eligible and consequently included in this meta-analysis. The search and selection process is depicted in details in Fig. 1.

The studies included in this meta-analysis were carried out in America [11–14, 16, 18], Germany [15], Turkey [17], and Ireland [19], and were published between 2000 and 2023. Of these, two were RCTs [11, 12], five were retrospective cohort studies [13–15, 17, 18], one cohort study had both prospective and retrospective components [19], and one was only available as a conference abstract and followed a retrospective cohort design [16]. The sample sizes for these trials varied from 100 to 868 patients, including a total of 4116 patients, with 2042 patients undergoing autologous blood patch intraparenchymal injection and 2,074 patients not receiving this treatment. In terms of methodological quality assessment, one RCT received a low-quality score [11], while the other received a high-quality score [12]. For the cohort studies, six studies received a high-quality score and had NOS scores ranging from 7 to 9 [13–15, 17–19]. The methodological quality of the cohort study presented in the form of a conference abstract is unclear [16]. Table 1 provides detailed characteristics and quality scores of the studies included in this meta-analysis.

**Table 1:** Characteristics and quality scores of included studies

NO	Author	Year	Country	Study design	Biopsy method	Guiding needle	Biopsy needle	Guidance method	Blood status	Blood patch group			Control group			Quality scores
										Patients	Age (years)	Lesion size (cm)	Patients	Age (years)	Lesion size (cm)	
1	Lang	2000	American	RCT	Aspiration biopsy	19G	20G–22G	Conventional CT	Clotted	50	54	2.0	50	48	2.4	3*
2	Malone	2013	American	RCT	Core biopsy	17G or 19G	18G or 20G	Conventional CT	Clotted	123	65	2.2	119	66	2.3	7*
3	Clayton	2016	American	Retrospective cohort study	Aspiration biopsy or core biopsy	19G	20G,22G, 23G	Conventional CT	Non-clotted	245	67	2.3	189	66	2.3	8#
4	Graffy	2017	American	Retrospective cohort study	Aspiration biopsy or core biopsy	17G-21G 65.6%19G	20G or21G	Fluoroscopy CT	Non-clotted	482	66.2	–	352	63.4	–	7#
5	Perl	2019	Germany	Retrospective cohort study	Core biopsy	13G, 15G, 17G,19G	–	Conventional CT	Non-clotted	419	–	–	449	63.4	–	7#
6	Turgut	2020	Turkey	Retrospective cohort study	Core biopsy	19G	20G	Conventional CT	Non-clotted	91	62.6	3.2	171	59.3	3.0	9#
7	Mendoza	2020	American	Retrospective cohort study	Core biopsy	–	99%20G	–	–	64	–	–	124	–	–	Unclear
8	Jain	2022	American	Retrospective cohort study	Core biopsy	17G or19G 98.5%19G	98.5%20G	Conventional CT	Clotted	309	69	–	227	68	–	8#
9	Duignan	2023	Ireland	Prospective and Retrospective cohort study	Core biopsy	19G	20G	Fluoroscopy CT	Non-clotted	259	68.4	2.4	393	68.1	2.8	7#

*RCT* Randomized-controlled trial, *G* Gauge

^*^Jadad scores, #NOS scores

### Pneumothorax

**Fig. 2:**
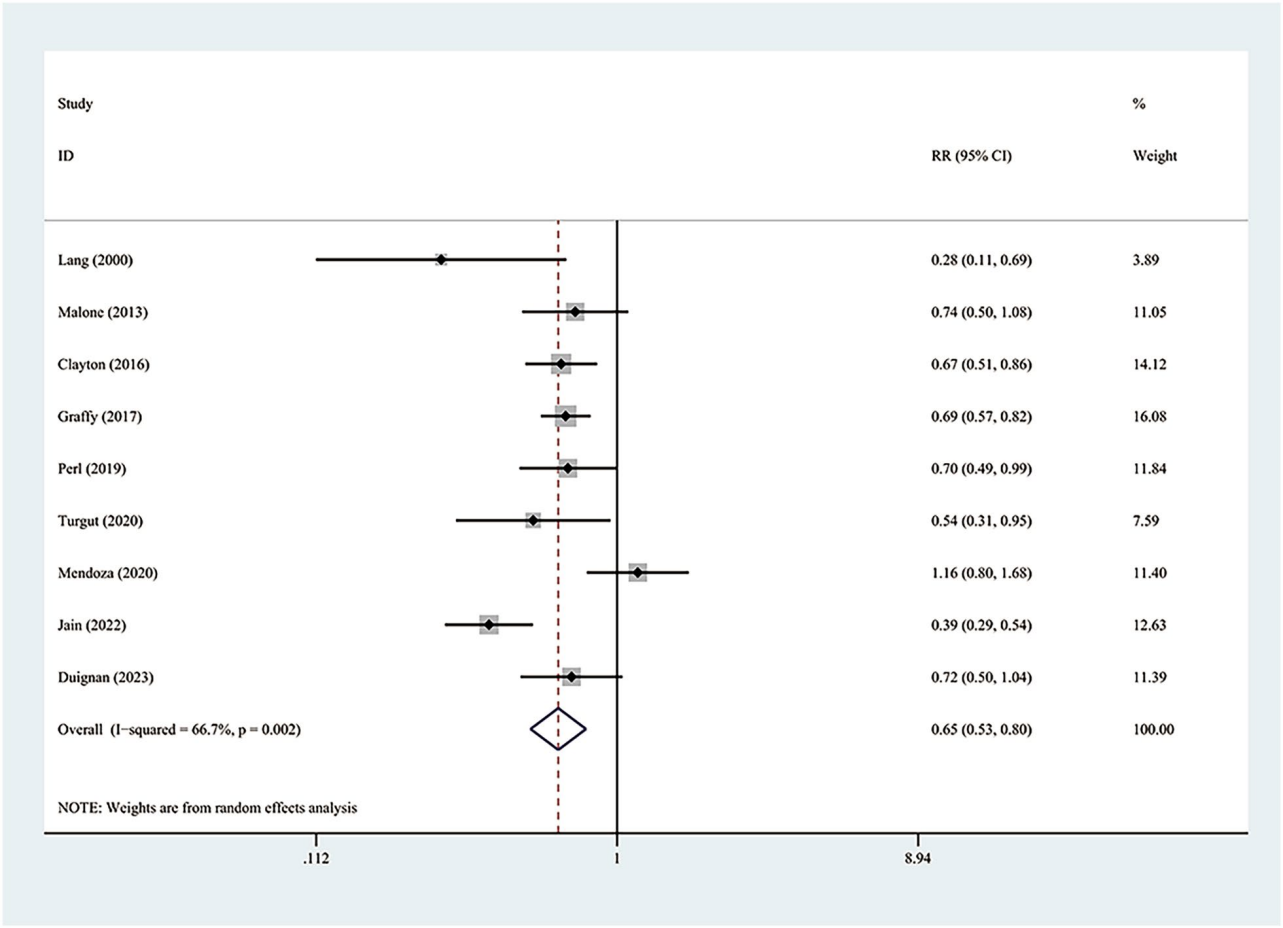
Forest plot describing the incidence of pneumothorax

**Fig. 3:**
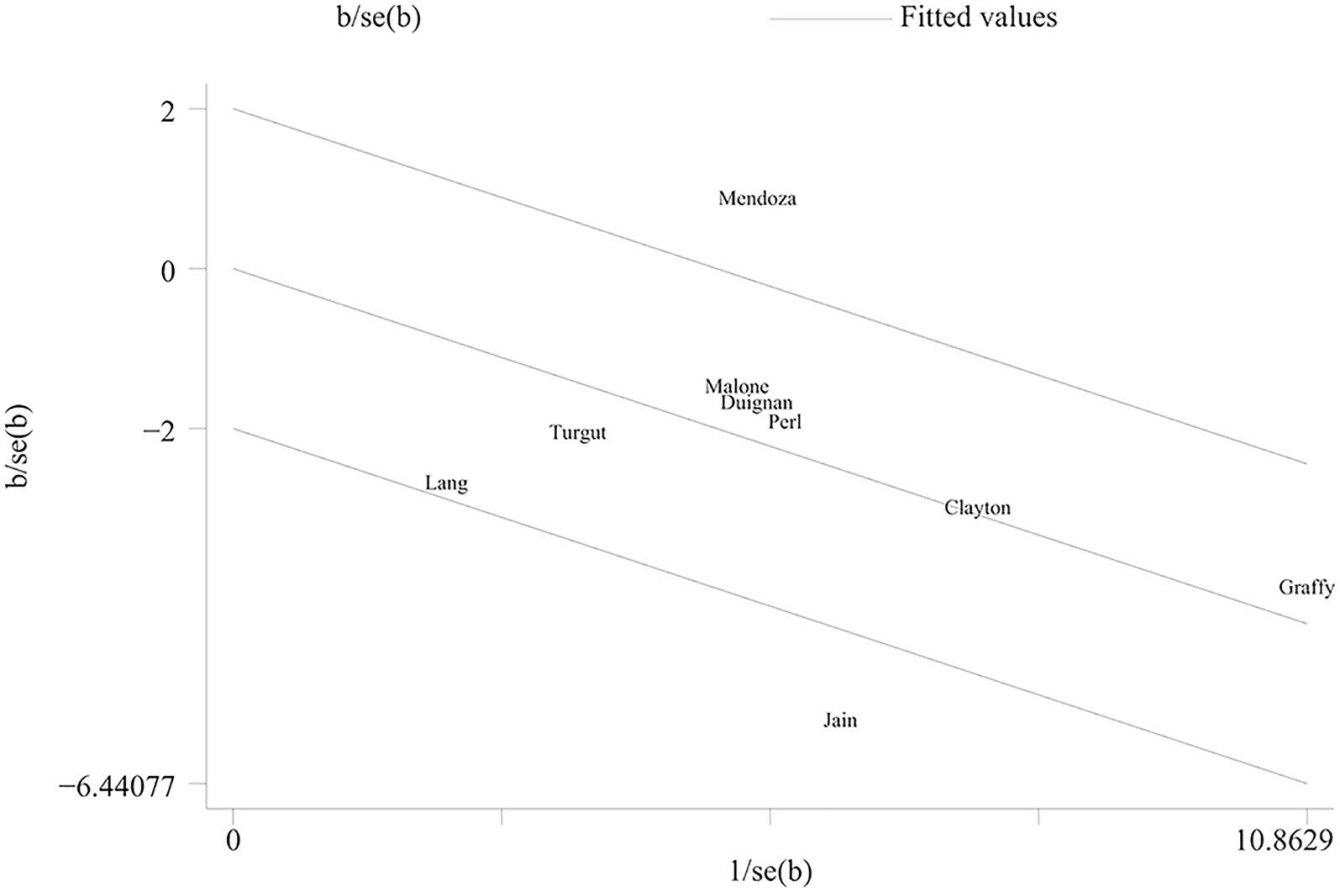
Heterogeneity analysis with a Galbraith plot

**Fig. 4:**
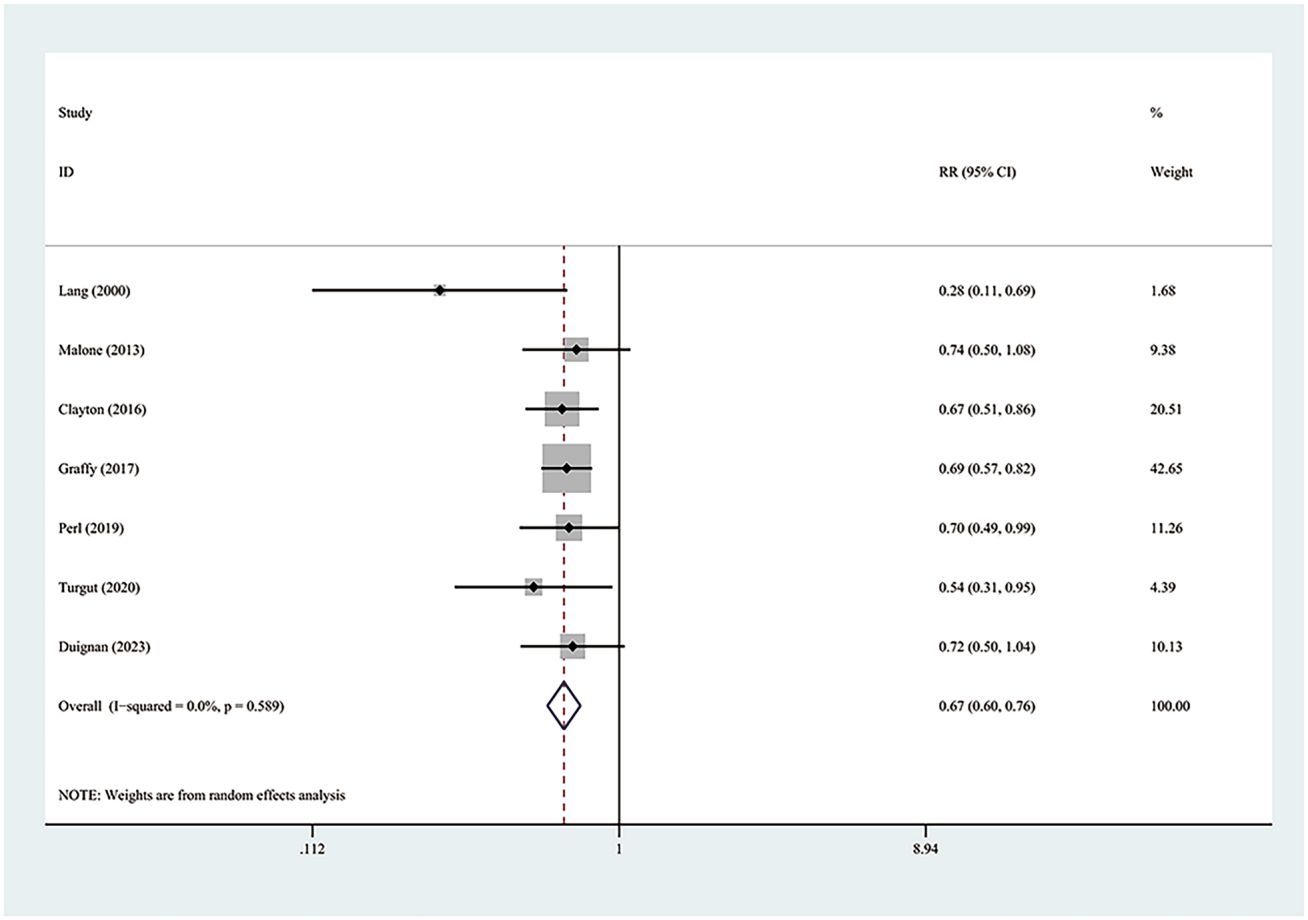
Forest plot describing the incidence of pneumothorax after removing the source of heterogeneity

The incidence of pneumothorax has been reported in the nine studies constituting this analysis. The amalgamated estimates verify that the autologous blood patch dramatically decreases the occurrence of pneumothorax (RR = 0.65; 95% CI 0.53–0.80; *P* = 0.00) (Fig. 2). However, considerable heterogeneity was observed (*I*^2^ = 66.7%; *P* = 0.002). The issue of inter-study heterogeneity was addressed through the creation of a Galbraith plot to visually tabulate the sources of this inconsistency (Fig. 3). Two outlier studies seen within the Galbraith plot constituted the main source of heterogeneity. Removing these studies brought about a significant decrease in heterogeneity (*I*^2^ = 0%, *P* = 0.589), and the direction of the new pooled effect size remained unchanged (RR = 0.67; 95% CI 0.60–0.76; *P* = 0.00) (Fig. 4). Further sensitivity analysis confirmed that the overall RR was stable and not affected by any single study (Additional file 1: Fig. S1). Both the funnel plot (Additional file 2: Fig. S2) and Egger’s test (*P* = 0.562) showed no significant publication bias.

### Chest tube placement

**Fig. 5:**
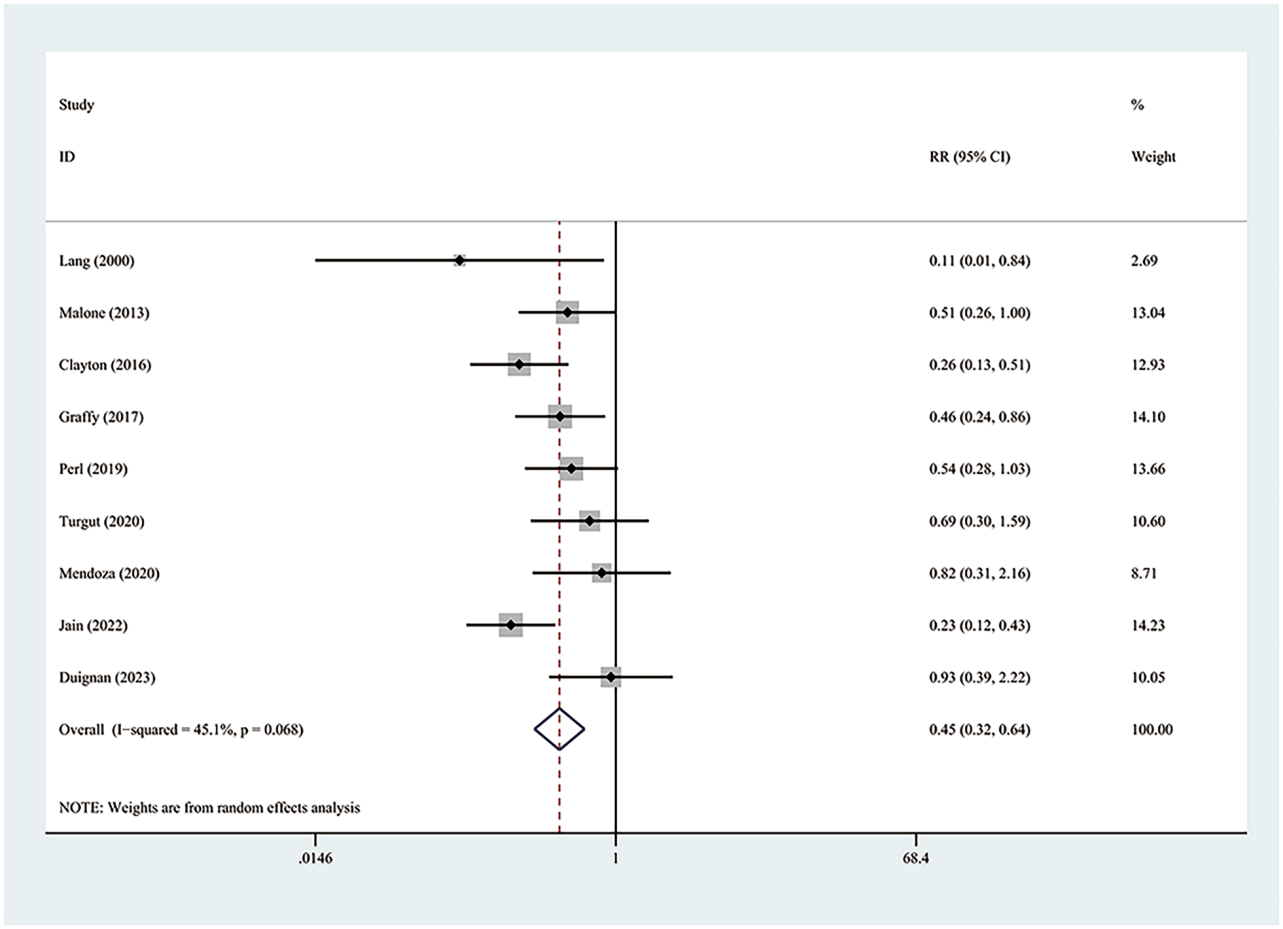
Forest plot describing the need for chest tube placement

Each of the nine studies encompassed in the meta-analysis reported chest tube placement due to pneumothorax. The collective findings indicate that implementing an autologous blood patch significantly reduces the number of instances that require this procedure (RR = 0.45; 95% CI 0.32–0.64; *P* = 0.00) (Fig. 5). Additionally, there was no significant heterogeneity observed (*I*^2^ = 45.1%; *P* = 0.07), as confirmed by the Galbraith plot (Additional file 3: Fig. S3). Sensitivity analysis demonstrated that the overall RR was stable and not influenced by any individual study (Additional file 4: Fig. S4). Both the funnel plot (Additional file 5: Fig. S5) and Egger’s test (*P* = 0.875) showed no significant publication bias.

### Subgroup analysis

Subgroup analysis was performed based on the study design, guidance method, and blood status, and the findings were presented in Table 2. Although the subgroup analysis did not elucidate the source of heterogeneity, the results suggest that the efficacy of an autologous blood patch is enhanced when conventional CT guidance and clotted blood are used.

**Table 2:** Results of subgroup analysis

Factors	Pneumothorax				Chest tube placement			
	Subgroup	RR(95%CI)	*P*	*I*^2^	Subgroup	RR(95%CI)	*P*	*I*^2^
Design	RCT (*n* = 2)	0.49(0.19–1.28)	0.15	74%	RCT(*n* = 2)	0.32(0.08–1.30)	0.11	51%
	Cohort study (*n* = 7)	0.67(0.54–0.83)	0.00	70%	Cohort study (*n* = 7)	0.47(0.31–0.70)	0.00	52%
Guidance method	Conventional CT (*n* = 6)	0.57(0.44–0.73)	0.00	58%	Conventional CT (*n* = 6)	0.38(0.25–0.57)	0.00	45%
	Fluoroscopy CT (*n* = 2)	0.69(0.59–0.82)	0.00	0%	Fluoroscopy CT (*n* = 2)	0.61(0.31–1.22)	0.16	42%
Blood status	Clotted (*n* = 4)	0.49(0.33–0.72)	0.00	62%	Clotted (*n* = 4)	0.37(0.19–0.71)	0.003	56%
	Non-clotted (*n* = 4)	0.69(0.60–0.78)	0.00	0%	Non-clotted (*n* = 4)	0.47(0.29–0.76)	0.002	46%

## Discussion

McCartney and his colleagues first introduced the blood patch technique in 1974 to mitigate the incidences of pneumothorax in lung biopsy [22]. Following this innovation, intermittent reports on its applications have surfaced. Moore reassessed the technique's efficacy using an equine model in 1995 [8]. His results demonstrated that the blood patch technique, whether executed in clotted or non-clotted form, could significantly increase the pressure threshold, reducing post-puncture pulmonary leakage in a typical laboratory lung environment. However, multiple clinical studies have witnessed inconsistent results, eschewing the technique from being recommended by established guidelines. Our current meta-analysis incorporated nine articles from various databases and included a total of 4,116 subjects. We sought to ascertain the effectiveness of the autologous blood patch in CT-guided lung biopsies. The data highlighted that the intraparenchymal injection of the autologous blood patch during the withdrawal of the guiding needle substantially mitigated the incidence of pneumothorax and the necessity for chest tube placement. This study, to the best of our knowledge, is the maiden meta-analysis providing holistic insights into this field.

In considering relative risk reduction, it is equally critical to pay attention to the absolutes of pneumothorax rates and chest tube insertion rates. The Society of Interventional Radiology (SIR) advises an upper limit of 45% for pneumothorax and 20% for chest tube insertion in lung biopsies [23]. On the other hand, the British Thoracic Society (BTS), through its 'Guidelines for Radiologically Guided Lung Biopsy’ [20], advocates for more stringent parameters, i.e., a maximum pneumothorax rate of 20.5% and a pneumothorax that requires chest drain insertion rate of 3.1%. The findings of our study indicate a noteworthy decrease in the overall incidence of pneumothorax, from 29.5% to 20.4%, alongside a corresponding reduction in the rate of chest tube insertion, from 9.4% to 4.0%, subsequent to the implementation of an autologous blood patch. Notably, the occurrence of pneumothorax falls below all recommended guidelines, while the chest tube insertion rate, although slightly higher than the British Thoracic Society (BTS) recommendation, remains significantly lower than the Society of Interventional Radiology (SIR) recommendation.

The technique provides multiple advantages. It allows for easy blood procurement without classifying as a transfusion. Furthermore, it does not necessitate specific processing and presents a minimal complication rate. While literature references convey a potential risk of systemic air embolism associated with this technique [19], our study did not assess this complication due to the lack of such occurrences reported in the included studies.

Substantial statistical heterogeneity was observed in the incidence of pneumothorax. The Galbraith plot analysis revealed that the Jain study [18] and the Mendoza study [16] were the sources of heterogeneity. In the Jain study, the control group had a higher percentage of patients with a history of chronic obstructive pulmonary disease (46.7% vs. 35.4%; *P* < 0.008), which may have contributed to the higher incidence rate of pneumothorax in this group. The Mendoza study was presented as a conference abstract, and it was not possible to assess whether there were any confounding factors that influenced the results.

The principal advantage of our research lies in the thoroughness and precision of our methodological approach. We meticulously complied with the PRISMA guidelines and employed two independent authors, working in a double-blind fashion. Our approach enhanced the inclusiveness of our systematic review while simultaneously decreasing the likelihood of missing pertinent publications. Furthermore, to mitigate possible data discrepancies, two separate authors carried out assessments for quality on each eligible trial and executed data extraction under double-blind conditions. Our meta-analysis only incorporates studies that strictly abide by our defined inclusion and exclusion criteria, thus enhancing the credibility and relevance of our findings. In addition, the allocation of a specific author, Yun Liu, who lacks a specialized background in lung biopsy for conducting this review, lessened potential bias in assessment of the studies. Her lack of prior engagement with these studies, coupled with her unbiased stance, assures a non-partisan evaluation.

This meta-analysis also has some limitations. Primarily, it has relied heavily on included retrospective cohort studies, a fact which could introduce selective bias. Additionally, the robustness of the research is impeded by the low-quality of an included RCT and the uncertain quality of a cohort study. Moreover, our subgroup analysis indicates that the effectiveness of autologous blood patches is amplified when applied with conventional CT guidance and clotted blood. However, the underlying reasons for these findings remain elusive. Therefore, we advocate for comprehensive, well-designed studies with larger population to delve into this matter further.

## Conclusion

Employing an autologous blood patch during the retrieval of the coaxial needle following a lung biopsy has shown significant efficiency in reducing both the incidence of pneumothorax and the need for chest tube placement.

### Supplementary Information

**Additional file 1. Fig. S1:** Sensitivity analysis of the incidence of pneumothorax.

**Additional file 2. Fig. S2:** Funnel plot of the incidence of pneumothorax.

**Additional file 3. Fig. S3:** Galbraith plot the need for chest tube placement.

**Additional file 4. Fig. S4:** Sensitivity analysis of the need for chest tube placement.

**Additional file 5. Fig. S5:** Funnel plot of the need for chest tube placement.

## Data Availability

The datasets used or analyzed during the current study are available from the corresponding author on reasonable request.
