# Supplementary figures and images for: Autologous blood patch intraparenchymal injection reduces the incidence of pneumothorax and the need for chest tube placement following CT-guided lung biopsy: a systematic review and meta-analysis
Source: Eur J Med Res. 2024 Feb 9;29:108. doi: 10.1186/s40001-024-01707-9 (PMC10854056; doi:10.1186/s40001-024-01707-9)

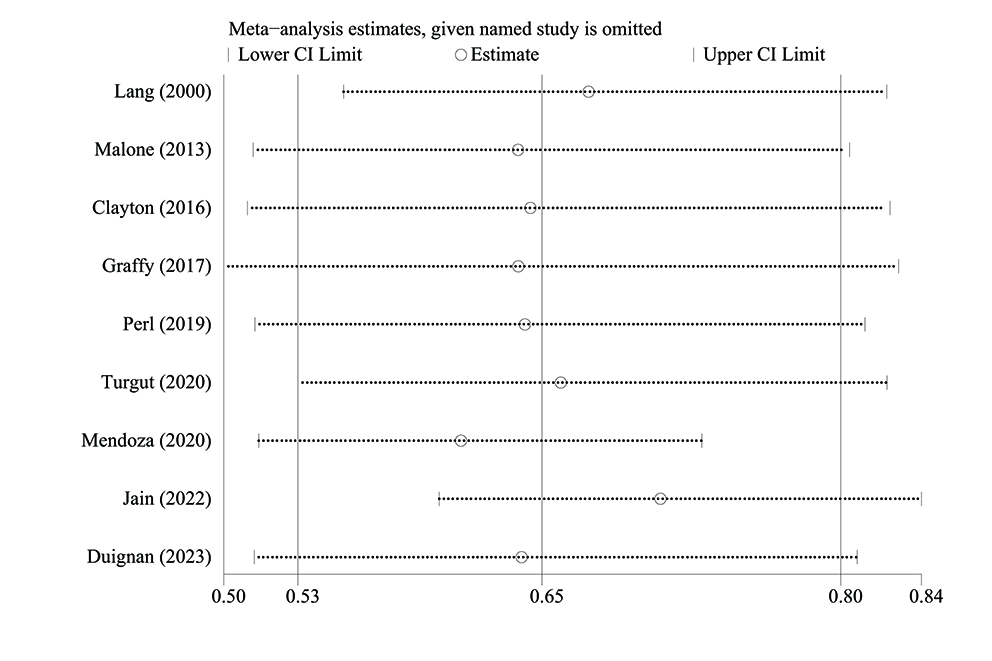

Supplement: Supplementary file 1 — Additional file 1. Fig. S1: Sensitivity analysis of the incidence of pneumothorax. [file 40001_2024_1707_MOESM1_ESM.tif]

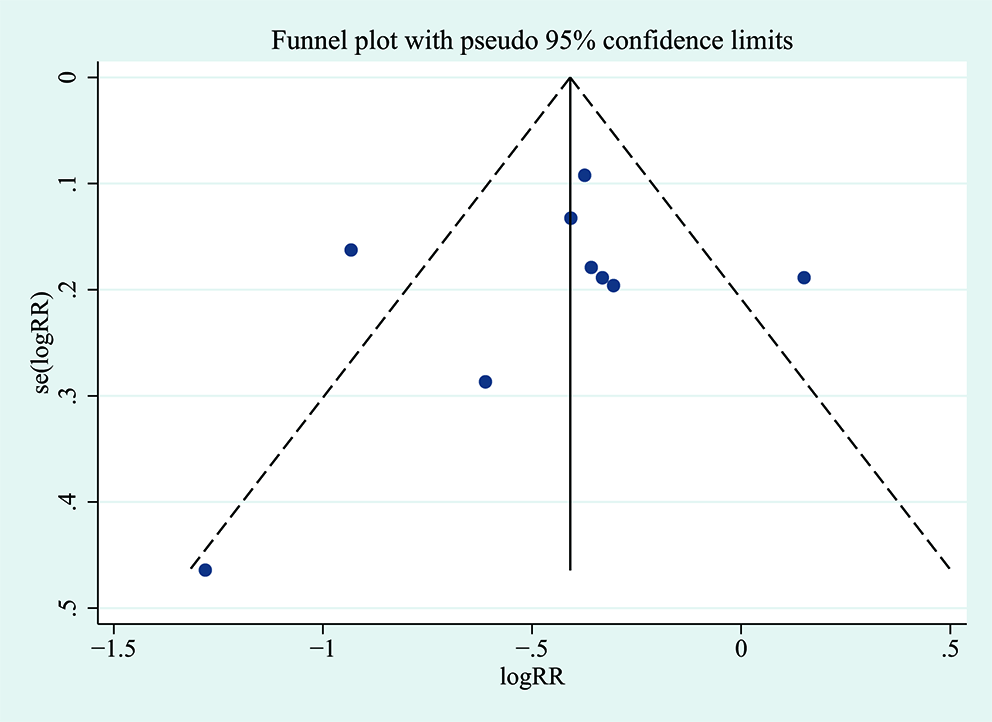

Supplement: Supplementary file 2 — Additional file 2. Fig. S2: Funnel plot of the incidence of pneumothorax. [file 40001_2024_1707_MOESM2_ESM.tif]

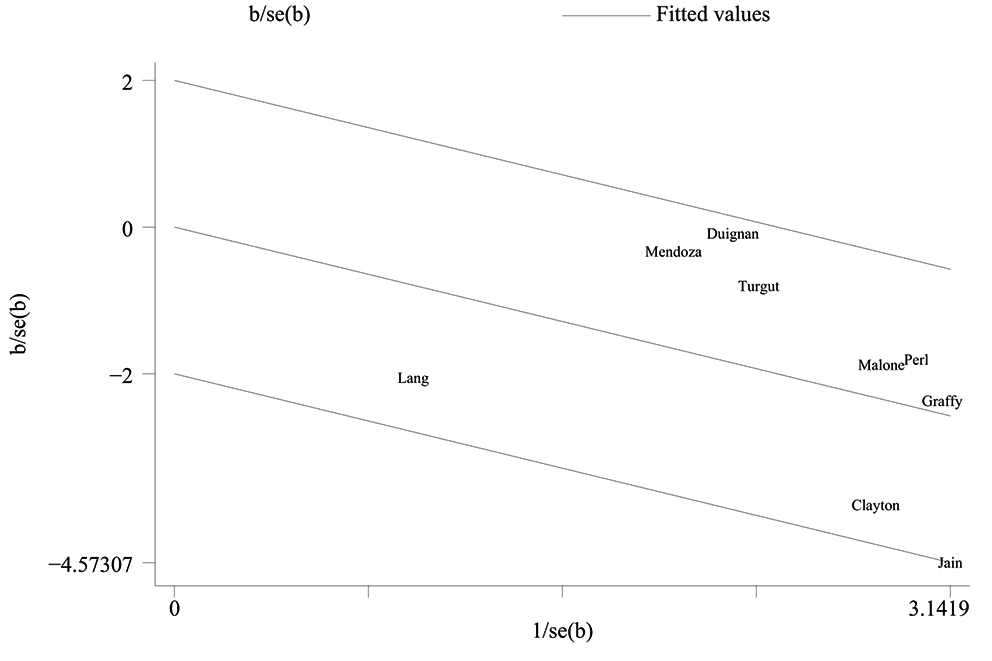

Supplement: Supplementary file 3 — Additional file 3. Fig. S3: Galbraith plot the need for chest tube placement. [file 40001_2024_1707_MOESM3_ESM.tif]

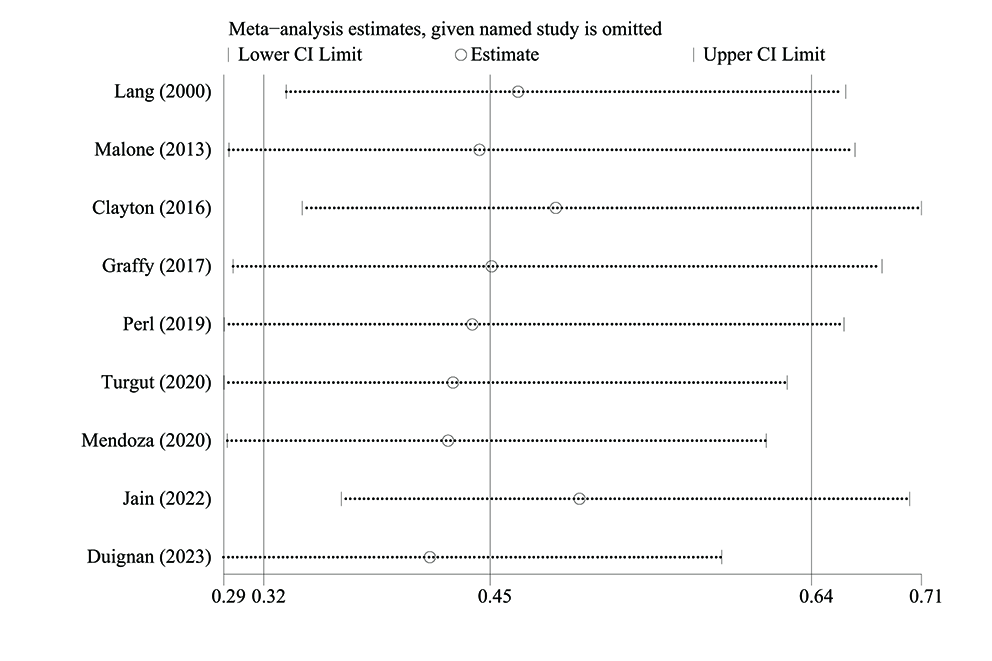

Supplement: Supplementary file 4 — Additional file 4. Fig. S4: Sensitivity analysis of the need for chest tube placement. [file 40001_2024_1707_MOESM4_ESM.tif]

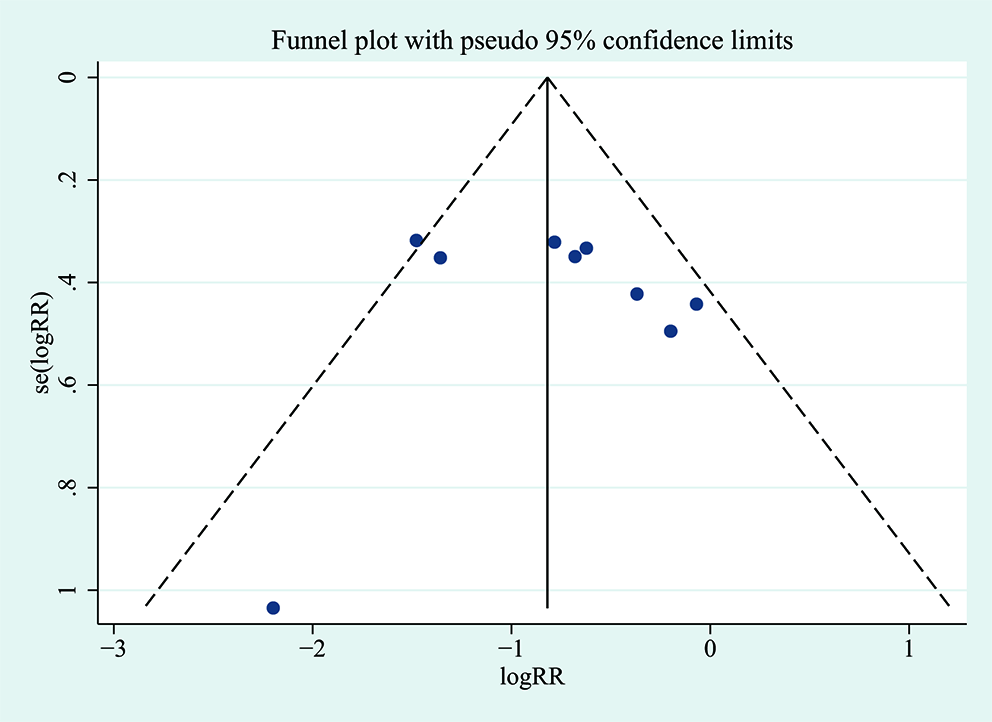

Supplement: Supplementary file 5 — Additional file 5. Fig. S5: Funnel plot of the need for chest tube placement. [file 40001_2024_1707_MOESM5_ESM.tif]
